# Hydrogel-Based Interfacial Solar-Driven Evaporation: Essentials and Trails

**DOI:** 10.3390/gels10060371

**Published:** 2024-05-27

**Authors:** Xiaoyun Hu, Jianfang Yang, Yufei Tu, Zhen Su, Qingqing Guan, Zhiwei Ma

**Affiliations:** 1 Key Laboratory of Oil and Gas Fine Chemicals Ministry of Education, College of Chemical Engineering, Xinjiang University, Urumqi 830017, China; huxiaoyun22@163.com (X.H.); yangjianfang1029@outlook.com (J.Y.); zhensu@xju.edu.cn (Z.S.); qingqing_guan@163.com (Q.G.); 2 School of Telecommunications and Intelligent Manufacturing, Sias University, Xinzheng 451150, China; 3 CAS Key Laboratory of Nano-Bio Interface, Division of Nanobiomedicine and i-Lab, Suzhou Institute of Nano-Tech and Nano-Bionics, Chinese Academy of Sciences, Suzhou 215123, China

**Keywords:** hydrogel, solar steam generation, desalination, wastewater treatment

## Abstract

Hydrogel-based interfacial solar-driven evaporation (ISDE) gives full play to the highly adjustable physical and chemical properties of hydrogel, which endows ISDE systems with excellent evaporation performance, anti-pollution properties, and mechanical behavior, making it more promising for applications in seawater desalination and wastewater treatment. This review systematically introduces the latest advances in hydrogel-based ISDE systems from three aspects: the required properties, the preparation methods, and the role played in application scenarios of hydrogels used in ISDE. Additionally, we also discuss the remaining challenges and potential opportunities in hydrogel-based ISDE systems. By summarizing the latest research progress, we hope that researchers in related fields have some insight into the unique advantages of hydrogels in the ISDE field and contribute our efforts so that ISDE technology reaches the finishing line of practical application on the hydrogel track.

## 1. Introduction

With the deepening global water scarcity crisis, turning to the ocean or wastewater for clean water is becoming a key solution [1,2,3]. While advancements in seawater desalination and wastewater treatment have been pivotal in securing freshwater sources, these methods come with their own set of challenges, including complexity, high energy consumption, and significant carbon emissions [4,5,6]. Solar-driven evaporation technology is emerging as a potentially valuable alternative, given its use of renewable, plentiful, and environmentally friendly solar energy to produce high-purity water through a simple water phase-change process [7,8,9,10]. In the meantime, solar-driven evaporation technology can be combined with bio-wastes to both mitigate the environmental impact of solid waste and to produce clean water more cost-effectively [11,12,13]. However, traditional solar-driven evaporation systems, despite their potential to meet the significant energy demands of up to 40 kJ·mol^−1^ required to evaporate water, face challenges with considerable heat loss when transporting heat to the evaporation position, leading to the inefficient use of solar energy and reduced evaporation efficiency [14,15]. Recent studies have demonstrated the potential to replace the traditional water-based heat transfer medium in solar-driven evaporation systems with nanofluids [16]. These nanofluids are engineered with high thermal conductivity to improve heat conversion efficiency and evaporation performance. However, there are urgent issues that need to be addressed regarding the application of nanofluids in solar-driven evaporation systems, including high cost, poor stability, environmental impacts, and sustainability concerns [17]. Therefore, the interfacial solar-driven evaporation (ISDE) system, which concentrates photothermal conversion and evaporation on the top air–water interface, significantly reduces thermal loss to the water bulk, and boosts water evaporation performance, has been proposed [18,19]. Furthermore, an increasing number of studies indicate that interfacial solar-driven evaporation systems not only serve the purpose of obtaining clean water but also have some extraordinary applications such as pathogen removal [20], extracting valuable substances from water [21], and obtaining electricity [22].

The performance of the ISDE system hinges on the strategic design and integration of photothermal materials and substrate materials [23,24], encompassing five pivotal stages: light absorption, heat conversion, water transport, heat conduction, and phase change, all of which are contingent upon the functionality of the materials involved [25]. Photothermal materials have high light absorption coefficients, which enable the efficient trapping of photons to initiate electron and nucleus modifications for photothermal conversion, a crucial process in heat acquisition for ISDE. Various photothermal materials, including metal nanomaterials (Ag, Au), semiconductor materials (TiO_2_), and carbon-based materials (carbon nanotubes), employ mechanisms like plasmonic resonance [26,27,28], electron–hole pair excitation and relaxation [29,30], and molecular thermal vibrations [31,32] to transform solar energy into thermal energy with over 95% efficiency, greatly improving the utilization of solar energy. Nevertheless, the efficiency of producing clean water from the thermal energy converted by photothermal material largely depends on the properties of the substrate material [33]. The substrate material serves as a structural support to improve the dispersion and thermal stability of the photothermal material. At the same time, light reflection could be reduced through surface topology design of the substrate material, thus ensuring the continuous and efficient operation of the photothermal material during the light absorption and heat conversion stage [34,35,36]. More importantly, at the stage of water transport and heat conduction, the substrate material plays a vital role. To minimize thermal loss to the water body and maximize the use of heat for evaporation, materials with low thermal conductivity are favored [37]. The substrate’s wettability, porous structure, and connectivity are crucial for ensuring even moisture distribution and efficient water transport to the upper photothermal layer, which directly affects the efficiency of heat transport and utilization during the evaporation process [38,39,40]. Additionally, it is of great importance that a substrate’s pollution resistance and mechanical integrity are taken into consideration, as these factors play a pivotal role in the long-term stability of the ISDE system, which can be achieved through meticulous surface engineering and structural design [41,42,43]. Therefore, the careful selection and design of the substrate materials are key to enhancing the evaporation performance of the ISDE system.

Hydrogels, with their unique three-dimensional network structure that exhibits solid-like characteristics, are the ideal flexible carriers for photothermal materials. They possess a high water content. Additionally, their adjustable physicochemical properties and diverse structures enrich and simplify the functionalization process compared to other substrate materials [44,45,46]. By strategically designing the hydrogel’s pore size, distribution, and hydrophilic or hydrophobic characteristics, it is possible to effectively regulate the state of water molecules within the hydrogel, reducing or eliminating the hydrogen bonding between large water clusters. This, in turn, lowers the enthalpy change necessary for water evaporation, accelerating the phase transition from liquid to gas [47,48]. Moreover, such design can also enhance the hydrogel substrate’s ability to continuously transport water to the top photothermal layer [49,50,51]. By deliberately engineering the polymer chains, hierarchical structures, or specific sites, hydrogels can attain remarkable heat resistance, pollution resistance [52,53], robust mechanical strength [54,55,56], and self-healing properties [57,58]. These attributes, in combination, guarantee their sustained stability throughout the evaporation process. Consequently, owing to their unparalleled structure and functionality, hydrogels exhibit a pronounced competitive edge as the preferred substrate material in ISDE systems.

**Figure 1:**
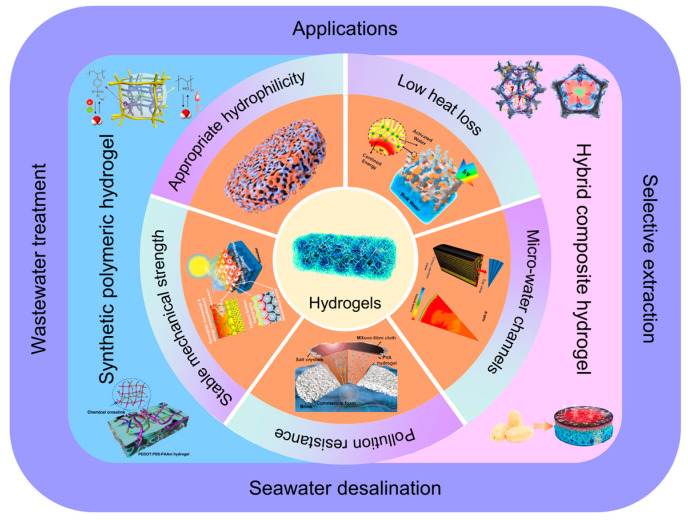
A schematic representation of the topics covered in this review. The schematic of hydrogels is reprinted with permission from ref [59]. Copyright 2020 Royal Society of Chemistry. Part two: design criteria of hydrogel substrates in ISDE systems, including appropriate hydrophily (reprinted with permission from ref [60]; copyright 2022 American Chemical Society), low heat loss (reprinted with permission from ref [61]; copyright 2019 American Chemical Society), micro-water channels (reprinted with permission from ref [62]; copyright 2023 Wiley-VCH Verlag GmbH), pollution resistance (reprinted with permission from ref [63]; copyright 2021 Wiley-VCH Verlag GmbH), and stable mechanical strength (reprinted with permission from ref [64]; copyright 2021 American Chemical Society). Part three: preparation and functional analysis of hydrogel substrates in ISDE systems. Reprinted with permission from ref [13,46,52,65]. Copyright 2020 Wiley-VCH Verlag GmbH. Copyright 2022 Elsevier. Copyright 2023 Wiley-VCH Verlag GmbH. Copyright 2023 Elsevier. Part four: applications of hydrogel-based ISDE systems.

This review comments on advancements in hydrogel substrate materials within the ISDE domain. Initially, it delves into the design criteria for hydrogel substrates in ISDE systems (Section 2), encompassing appropriate hydrophilicity, low thermal loss, micro-water channels, pollution resistance, and stable mechanical strength (Figure 1). Following this, the review methodically details the preparation and distinctive properties of hydrogel substrate materials (Section 3), emphasizing their practical efficacy in applications such as seawater desalination, wastewater treatment, and selective extraction (Section 4). In the final segment of the review, it explores the potential opportunities and challenges that hydrogel-based ISDE systems encounter (Section 5). Compared to other reviews, this review offers a comprehensive assessment of hydrogel-based ISDE systems, covering design principles, preparation methods, performance requirements, and their extensive applications from multiple perspectives. It provides researchers with a detailed guide to understanding the unique advantages of hydrogels in the ISDE field and their advancement towards practical applications.

## 2. Design Criteria of Hydrogel Substrates in ISDE Systems

The evaporation rate (*r*) and efficiency (*η*) serve as crucial metrics for evaluating the ISDE system’s performance [66,67,68]. The evaporation rate (*r*) measures the mass change rate of evaporable water per unit area, defined by the formula:

*r* = Δ*m*/(*S*·*t*) (1)

where Δ*m* denotes the mass of water vapor generated through evaporation, *S* signifies the effective evaporation area, and *t* represents the time of irradiation. This formula clearly reflects the ISDE system’s capability to produce clean water.

The evaporation efficiency (*η*), representing the conversion rate of solar energy into water vapor energy, indicates the amount of solar energy utilized in generating water vapor at the evaporation interface. The formula for calculating *η* is:

*η* = *q_evap_*/*q_in_* = (Δ*m_sunlight_* − Δ*m_dark_*) *h_vap_*/(*C_opt_*·*P_0_*) (2)

where *q_evap_* is the evaporation energy, *q_in_* is the total solar energy received, Δ*m_sunlight_* is the apparent evaporation rate, Δ*m_dark_* is the evaporation rate in the dark, *h_vap_* is the total enthalpy change for water evaporation, *C_opt_* is the optical concentration on the absorber’s surface, and *P*_0_ is the standard solar radiation, set at 1 kW·m^−2^. The evaporation efficiency (*η*) measures the capacity for solar energy conversion into heat, a critical step in triggering the ISDE system.

The hydrophilicity, thermal conductivity, and water transport channel design of the hydrogel substrates play a significant role in reducing water evaporation enthalpy, enhancing thermal efficiency, and stabilizing water supply at the evaporation interface [69,70,71]. Achieving the optimal balance among these properties is crucial for rapid and efficient evaporation. Moreover, the pollution resistance and mechanical stability of the hydrogel substrates ensure the long-term stable operation of high-performance ISDE systems [55,72,73]. This section not only explores the essential guidelines for designing high-performance hydrogel substrates for ISDE but also lays a solid foundation for the future development of high-performance ISDE systems that incorporate many advantages by finding the optimal balance among these principles (Table 1).

### 2.1. Appropriate Hydrophilicity

The hydrophilicity of hydrogels is crucial for the performance of ISDE systems, directly affecting the evaporation rate and efficiency. The hydrophilicity of hydrogels originates from the abundant hydrophilic functional groups on their polymer chains [74], such as hydroxyl, amino, and carboxyl groups. These functional groups are adept at drawing water molecules into the hydrogel’s three-dimensional network, where they exhibit varying diffusion behaviors influenced by the network’s compositional and structural nuances [75,76].

**Figure 2:**
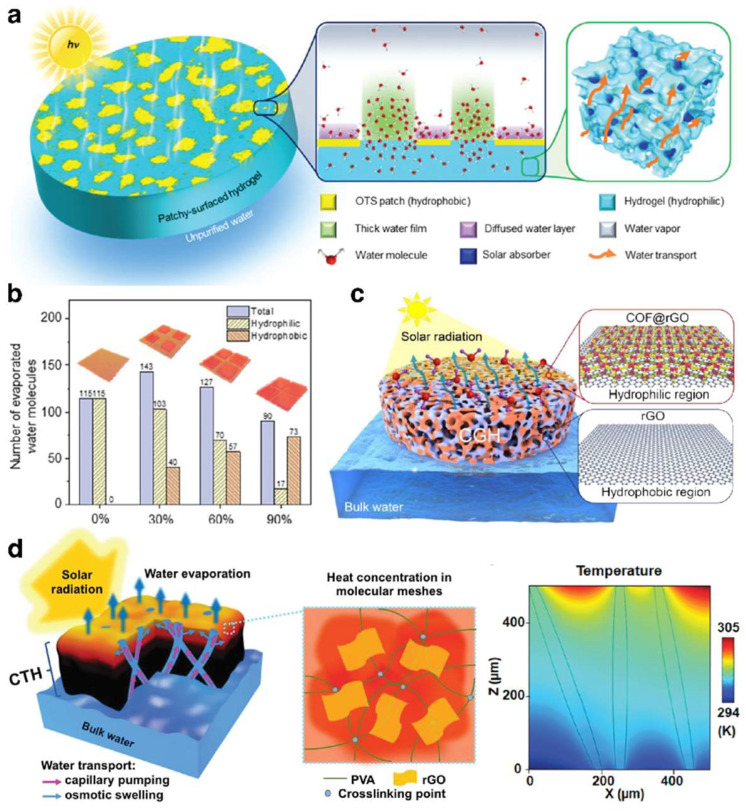
(**a**) The scheme of the synergy between PVA’s hydrophilic zones and OTS’s hydrophobic islands within hydrogel for accelerated water release. (**b**) The relationship between the number of evaporated water molecules and the hydrophilicity of hydrogels. (**a**,**b**) Reprinted with permission from ref [77]. Copyright 2020 Royal Society of Chemistry. (**c**) The scheme of the COF/graphene composite dual-zone hydrogel with optimized hydrophilic zones for efficient evaporation. Reprinted with permission from ref [60]. Copyright 2022 American Chemical Society. (**d**) The scheme of hydrogel with appropriate hydrophilicity to balance water transport and thermal loss for effective evaporation. Reprinted with permission from ref [24]. Copyright 2018 Royal Society of Chemistry.

Firstly, the unique interaction within the hydrogel network imparts water molecules in hydrogels with distinct characteristics, setting them apart from those in bulk water. Specifically, water molecules in bulk water can form large clusters through hydrogen bonding, owing to their dipolar nature. This means that transforming water molecules in bulk water into gas involves overcoming the strong forces of hydrogen bonds, necessitating a significant amount of energy, i.e., a high evaporation enthalpy. Conversely, water molecules within hydrogels can not only cluster through intrinsic hydrogen bonding but also engage in multifaceted interactions with the hydrophilic functional groups on the polymer chains, presenting three discernible states: free water, intermediate water, and bound water. By strategically adjusting the balance and distribution of hydrophilic and hydrophobic components in the hydrogel, the mutual binding of water molecules can be effectively reduced, increasing the proportion of intermediate water that evaporates more easily, thereby significantly improving the evaporation rate [47,49]. Yu et al. [77] developed the ISDE system using a hydrophilic polyvinyl alcohol (PVA) hydrogel substrate partially modified with trichloro(octadecyl)silane (OTS), illustrating this design principle effectively (Figure 2a). This innovative hydrogel structure featured a synergistic relationship between PVA’s hydrophilic zones and OTS’s hydrophobic islands, leading to rapid water evaporation by increasing hydrophobic areas to concentrate water in hydrophilic zones, thereby thickening the water layer and lowering the evaporation barrier for faster water release. Nonetheless, it was crucial to recognize that an excessive presence of hydrophobic islands could undermine the hydrogel substrate’s natural hydrophilicity, potentially slowing down the evaporation process (Figure 2b). This underscores the vital balance between the hydrophilicity of the hydrogel substrate and its evaporation efficiency. Thomas et al. [60] developed a covalent organic framework (COF)/graphene composite dual-zone hydrogel substrate, which significantly improves light absorption and water retention and decreases the enthalpy required for water evaporation. This enhancement was achieved by carefully managing the distribution of hydrophilic COF-loaded reduced graphene oxide (COF@rGO) and hydrophobic reduced graphene oxide (rGO) zones (Figure 2c).

Moreover, the high hydrophilicity of the hydrogel substrate endows it with exceptional water absorption capabilities, facilitating the movement of water from the base of the ISDE system to the photothermal layer on the surface, thereby enhancing the evaporation rate [24] (Figure 2d). However, elevated hydrophilicity may lead to increased water content within the polymer network, potentially hastening the thermal conduction of surface heat to the adjacent water, resulting in heightened thermal loss. This indicates the necessity to find an optimal balance between the hydrogel substrate’s hydrophilicity and its evaporation efficiency. Therefore, to achieve a high evaporation efficiency and rate in the ISDE system, it is imperative to moderately control the hydrophilicity of the hydrogel substrate to ensure low evaporation enthalpy and swift water transport.

### 2.2. Low Heat Loss

**Figure 3:**
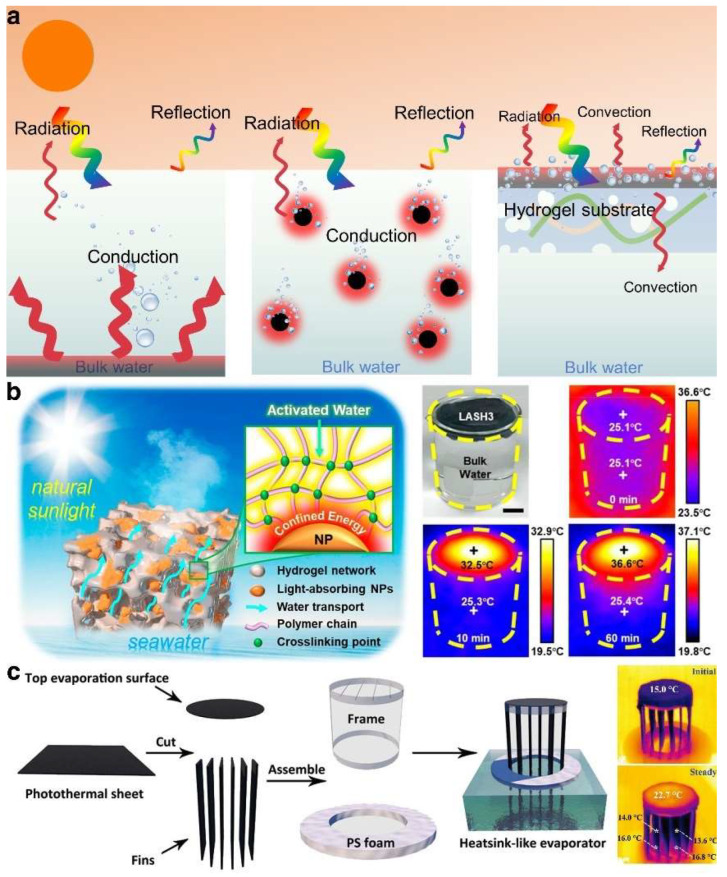
(**a**) The scheme of water vapor generation and heat transport in three typical ISDE systems. (**b**) Reduced heat loss of ISDE system due to energy restriction at the polymer–nanoparticle interface and the low thermal conductivity of polyvinyl alcohol. Reprinted with permission from ref [61]. Copyright 2019 American Chemical Society. (**c**) The scheme of a novel hydrogel with fin structures that separate the photothermal material and the water as well as regulate thermal convection to reduce heat loss. Reprinted with permission from ref [79]. Copyright 2021 Wiley-VCH Verlag GmbH.

Addressing thermal losses due to heat radiation, convection, and conduction, traditional solar-driven evaporation systems employing bottom and overall heating methods exhibit a significantly low solar energy utilization rate, with an evaporation efficiency of merely 40% [18,19] (Figure 3a). Conversely, the ISDE system, which uses a substrate material for support and concentrates photothermal material at the top air–water interface, minimizes thermal dissipation into the water body by focusing heat. It also accelerates the interface temperature response through localized heating technology, thereby shortening the steam generation time and substantially enhancing evaporation efficiency. This demonstrates that managing thermal loss is essential for achieving high evaporation performance.

Hydrogels emerge as exemplary substrates for the ISDE system due to their distinctive three-dimensional network structure and copious functional groups, facilitating the binding of various photothermal materials. Heat transport in hydrogels through free water (also known as bulk water) with a relatively high thermal conductivity (0.5 W·m^−1^·K^−1^) is one of the main pathways for heat loss. Notably, hydrogels derived from materials with low thermal conductivity, such as polyurethane, polystyrene, or fibers, can be used to prepare low-thermal-conductivity hydrogel substrates that effectively segregate photothermal materials and free water, concentrating more heat on the ISDE system’s surface and thereby markedly reducing thermal loss. Yu et al. [61] reported a sponge-like hydrogel composed of Ti_2_O_3_ nanoparticles and polyvinyl alcohol, which, owing to the energy restriction at the polymer–nanoparticle interface and the low thermal conductivity of polyvinyl alcohol, efficiently mitigated thermal loss (Figure 3b). Furthermore, the thermal conductivity of air, at only 0.026 W·m^−1^·K^−1^, is significantly lower than that of water. This discrepancy has led researchers to innovate by constructing a sealed, air-filled cavity between the photothermal material and the ambient water, aiming to further mitigate heat loss [78]. In a more ingenious design, a channel, narrower than the bottom water supply pipe, was introduced between these two components [79] (Figure 3c). This design not only preserves the air layer to prevent direct contact between the photothermal material and water but also effectively interrupts the pathway for thermal convection from the hot water to the surrounding cooler water regions. Such a strategy underscores that by meticulously designing the internal network structure to regulate the direction and speed of water transport, it is feasible to significantly curtail heat transport induced by thermal convection, thus minimizing heat loss.

From an energy management standpoint, as previously elucidated, the hydrogel’s moderate hydrophilicity, through augmenting the volume of intermediate water within the network, can efficiently decrease the energy requisite for the evaporation process, serving as a viable means of offsetting heat loss (Figure 2d). More notably, enhancing hydrophilicity elevates the proportion of bound water in the gel network. This elevation impedes heterogeneous ice nucleation within the hydrogel, bolsters the ISDE system’s resistance to freezing, and effectively safeguards the photothermal system against potential damage in cold environments, thereby fulfilling the objective of minimizing heat loss from another dimension [80].

### 2.3. Micro-Water Channels

The design of water channels is pivotal in managing water transport from the lower to the upper heating layer. When the water transport rate surpasses the evaporation rate at the evaporation interface, it results in an excessive accumulation of water, leading to the heat generated by the top photothermal layer being absorbed and dissipated by the surplus water, consequently diminishing the evaporation efficiency [71,81]. On the contrary, if the water transport rate falls below the evaporation rate at the interface, the water content becomes insufficient, hampering the sustainability of the evaporation process, thereby decreasing water vapor production [34]. Hence, aligning the water transport rate with the evaporation rate at the interface is one of the key challenges in optimizing evaporation performance, and water channel design is an important aspect of addressing this challenge.

As mentioned above, the hydrophilic three-dimensional network structure of hydrogels plays a significant role in attracting water molecules and facilitating their diffusion. Engineering polymer monomers or functionalized polymer chains enables the fine-tuning of hydrogels’ hydrophilicity and three-dimensional network, allowing for precise control of the water transport rate [33]. Moreover, capillary action significantly contributes to water movement within hydrogel channels. Utilizing the adjustable physicochemical properties and morphology of hydrogels, by precisely tuning the pore size and distribution to construct micro/nano or multi-level channels, it is possible to effectively control the water transport rate and the interface evaporation rate, thus enhancing the evaporation efficiency. Inspired by the radial arrangement of microchannels in coniferous trees and the geometric shapes of vertical container structures, Yu et al. [62] developed a 3D sponge-like hydrogel-based ISDE system with an adjustable porous structure, which significantly enhanced water transport capacity by nearly two orders of magnitude while maintaining a high energy conversion efficiency of 95%. Similarly, Gao et al. [82] engineered a composite hydrogel-based ISDE system using 2D woven fabric, which, thanks to the high water content and hierarchical porous structure of the composite hydrogel, exhibited a high evaporation rate. Additionally, Gao et al. explored the impact of varying hydrogel thicknesses (2, 4, 6, and 10 mm) on the evaporation rate, found that increasing the hydrogel’s thickness extended the water transport path, potentially causing the water transport rate to lag behind the evaporation rate, limiting the water surface evaporation rate and thus reducing the evaporation rate from 2.49 kg·m^−2^·h^−1^ at a thickness of 2 mm to 1.90 kg·m^−2^·h^−1^ at a thickness of 10 mm.

### 2.4. Pollution Resistance

The pollution resistance capability of hydrogel substrates is crucial for the stable operation of ISDE systems. During the process of extracting clean water from seawater or wastewater using ISDE systems, water molecules move through the water channels of the hydrogel and evaporate at the surface interface. Meanwhile, the hydrogel’s attraction to pollutants causes these substances to enter the water channels, forming concentration gradients. This challenge is particularly significant for saline water bodies, as the repeated evaporation process may lead to excessive accumulation of salt ions at the evaporation interface, forming salt crystals [83]. Such accumulation could not only potentially block the light absorption layer, hindering the penetration of sunlight and reducing the system’s effective evaporation area, but also clog the water channels, restricting water flow and greatly reducing evaporation efficiency. Therefore, enhancing the salt rejection capability of hydrogels is key to improving their pollution resistance performance.

**Figure 4:**
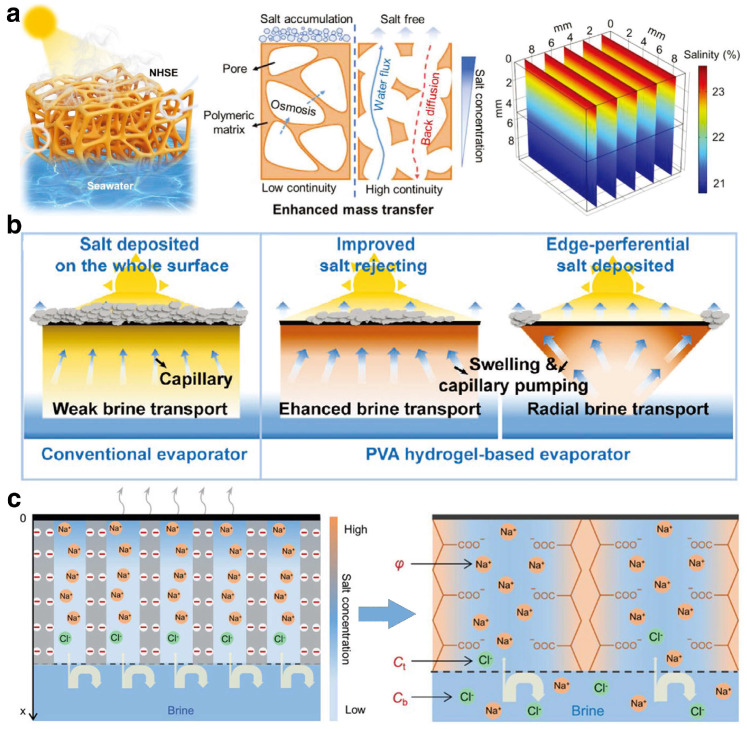
(**a**) The scheme of a high-porosity nanofiber-based hydrogel with a continuous microchannel structure for preventing salt accumulation. Reprinted with permission from ref [84]. Copyright 2023 Wiley-VCH Verlag GmbH. (**b**) The PVA hydrogel featuring an inverted three-dimensional conical structure induces edge-preferential salt deposited. Reprinted with permission from ref [63]. Copyright 2021 Wiley-VCH Verlag GmbH. (**c**) The scheme of salt-resistance by leveraging electrostatic forces to disrupt the interactions between salt ions. Reprinted with permission from ref [86]. Copyright 2021 Wiley-VCH Verlag GmbH.

An effective strategy to address this challenge is by diluting the salt concentration at the evaporation interface. Xu et al. [84] developed a high-porosity nanofiber-based hydrogel substrate with a continuous microchannel structure to promote rapid water transport to the evaporation interface, achieving efficient mass transfer and thus preventing salt accumulation (Figure 4a). However, due to contact with free water with higher thermal conductivity, the thermal energy at the evaporation interface might dissipate, affecting evaporation efficiency. To overcome this issue, Li et al. [85] designed a hydrogel substrate with a Janus structure, featuring a hydrophilic bottom and a hydrophobic surface. The hydrophobic top layer acts as a barrier to prevent salt deposition in the light absorption layer, while the hydrophilic channels at the bottom efficiently redirect high-concentration saltwater back into the bulk water, thereby demonstrating superior salt rejection performance. Additionally, strategically managing the accumulation areas or migration paths of salt is also an effective strategy to reduce salt accumulation at the evaporation interface. Tang et al. [63] utilized polyvinyl alcohol (PVA) hydrogel to develop an ISDE system with an innovative inverted three-dimensional conical structure, enhancing the vertical radial transport of saltwater (Figure 4b). Due to the extended transport distance, the movement of salt to the edge regions is inherently slower, resulting in salt progressively accumulating and preferentially crystallizing at the edge regions during evaporation. This strategic process effectively shields the top evaporation interface from direct salt contamination. Lastly, the nucleation and crystallization barrier of salt can be elevated through the use of polyelectrolyte hydrogels, which contain a high concentration of negatively charged functional groups [86] (Figure 4c). This improvement boosts the salt rejection capabilities of hydrogels by leveraging electrostatic forces to disrupt the interactions between salt ions.

### 2.5. Stable Mechanical Strength

The stable mechanical strength of the hydrogel substrate is a key factor to consider when designing an ISDE system. In the process of extracting clean water from seawater or wastewater using the ISDE system, the hydrogel substrate not only needs to efficiently transport water molecules and promote their evaporation at the surface interface, but it also must withstand continuous solar radiation and physical and chemical stresses caused by environmental changes [87]. Thus, maintaining the structural and functional integrity of the hydrogel substrate is a core element in developing an efficient ISDE system.

Hydrogels’ distinctive properties, such as their resistance to melting or dissolving, are mainly attributed to their three-dimensional crosslinked network formed through chemical bonds or physical interactions between polymer chains [75]. This complex structure evenly disperses external forces, minimizing localized stress concentrations, which is a key factor in enhancing the mechanical stability of hydrogels. Modifying the type and concentration of crosslinkers to enhance the crosslinking density within the hydrogel network proves to be an efficient strategy for bolstering its mechanical robustness and adaptability to diverse environmental challenges. Zong et al. [64] developed a polyvinyl alcohol (PVA)-based composite hydrogel with a highly cross-linked network, which exhibited exceptional toughness (~231 kJ m^−2^) and ultimate strain (~310%), ensuring a stable evaporation rate of 4 kg·m^−2^·h^−1^ with long-term exposure to solar radiation under 1 sun over one week. However, it is important to note that an overly high crosslinking density might reduce the toughness of the hydrogel and affect the efficiency of water transport. Therefore, finding a balance to ensure that water transport efficiency is not compromised while increasing the crosslinking density is an important consideration. The design of the crosslinking method is another way to improve the mechanical strength and stability of hydrogels, primarily involving two types: physical and chemical crosslinking [44]. Chemical crosslinking, through the formation of covalent bonds, usually creates a more stable network structure, thereby enhancing the strength and toughness of hydrogels. Additionally, hydrogels based on dynamic covalent bonds [88,89] (such as imine, disulfide, hydrazone, and boronate ester complexes) have been developed. These hydrogels can trigger self-healing properties through external stimuli (such as light, heat, pH, and electricity) [90], further enhancing the functional stability and lifetime of hydrogels in ISDE systems. In contrast, physical crosslinking mainly relies on intermolecular interactions, such as hydrogen bonds and hydrophobic interactions, which are relatively less stable. However, the dynamic nature of non-covalent interactions in physical crosslinking allows it to be integrated with chemical crosslinking networks. This integration helps dissipate energy through deformation, breakage, or slippage under stress, thereby improving the hydrogel’s resistance to cracking and fatigue while endowing it with self-healing capabilities [91]. A hydrogel developed by Xu et al. [92], made from polyvinyl alcohol (PVA), sodium alginate (SA), and sodium polyacrylate (PAAS), utilizes strong hydrogen bonding along with dynamic cross-linking points, demonstrating stable mechanical strength with a tensile strength of 25.57 kPa and an elongation at break of 754.8%. Additionally, the hydrogel exhibits satisfactory self-healing capabilities, irrespective of whether it is exposed to air, fresh water, or a simulated sewage environment.

## 3. Preparation and Functional Analysis of Hydrogel Substrates in ISDE Systems

Hydrogel substrates are essential, integrating efficient photothermal materials and precisely managing water transport, thermal conductivity, pollution resistance, and durability, all contributing to exceptionally high evaporation efficiency in ISDE systems. Despite this, skillfully balancing these diverse functionalities to meet the five major design criteria remains a focal point of current research. Utilizing the adjustable physicochemical properties and variable forms of hydrogels, researchers have significantly advanced the development of hydrogel substrates that enhance the performance of ISDE systems in multiple aspects simultaneously. This section explores the fabrication techniques of these substrates and their direct impact on performance, highlighting their potential to foster breakthroughs in solar evaporation technology. These findings are vital for further advancements in material innovation and optimization, indicating the ability of hydrogel-based ISDE systems to increasingly address global water shortages.

### 3.1. Synthetic Polymeric Hydrogels

**Figure 5:**
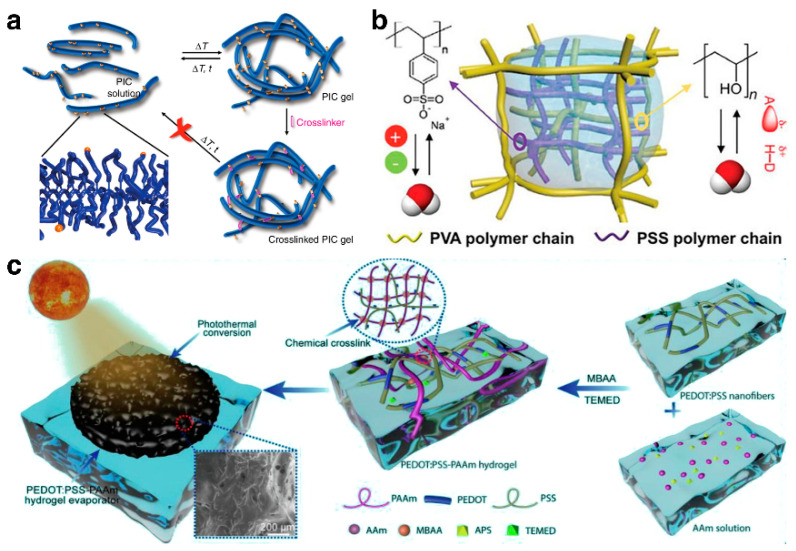
(**a**) The crosslinking method of hydrogels. Reprinted with permission from ref [95]. Copyright 2018 Springer Nature. (**b**) The interpenetrating double-network hydrogel-based ISDE system achieving simultaneously management of water and thermal energy. Reprinted with permission from ref [46]. Copyright 2020 Wiley-VCH Verlag GmbH. (**c**) Design of PEDOT: PSS-PAAm double-network hydrogel substrate for stable and efficient evaporation. Reprinted with permission from ref [56]. Copyright 2022 Elsevier.

Hydrogel technology has attracted attention due to its abundant raw material sources [93]. Numerous natural polymers, such as cellulose, alginate, and hyaluronic acid, boast long molecular chains and rich functional groups, which can form hydrogels with high hydrophilicity and excellent biocompatibility, either through inherent physical actions or by the addition of cross-linking agents [94]. The formation mechanisms of these hydrogels mainly involve creating physical cross-links or covalent bonds between polymer chains [95] (Figure 5a). To better control key properties such as molecular weight, hydrophilicity/hydrophobicity, or environmental tolerance, numerous synthetic monomers and modified polymers, including acrylamide, methyl methacrylate, carboxymethyl cellulose, and polyvinylpyrrolidone, have been developed [44]. By adjusting the cross-linking density and degree of polymerization of the polymers, their key performance in water transport, thermal conductivity, and mechanical strength can be further optimized [33].

While ISDE systems based on single-network hydrogels exhibit commendable evaporation efficiency and stability in practical uses, precisely managing their physical and chemical properties to suit specific application demands remains a challenge. Furthermore, traditional single-network hydrogels fall short of fully satisfying the five core principles of contemporary design. Consequently, ISDE systems based on multi-network hydrogels, which enhance the multifunctionality of hydrogels through the combined benefits of various networks, have emerged as a focal point of research. The hydrogel created by Yu et al. [46], integrating a polystyrene sulfonate (PSS) network with a densely cross-linked polyvinyl alcohol (PVA) network, effectively manages both water and thermal energy simultaneously (Figure 5b). Notably, the ionic polymer PSS engages in electrostatic interactions with water molecules, activating over 50% of the water into an intermediate water state, thus drastically reducing the energy required for evaporation at solar interfaces. Simultaneously, the robust PVA network tightly controls the water content within the hydrogel, minimizing energy loss and boosting efficiency. This cooperative interaction enables the hydrogel to achieve an impressive evaporation rate of 3.86 kg·m^−2^·h^−1^ and an energy efficiency of 92%. On another note, the hydrogel designed by Lu et al. [56] is crafted by merging polystyrene sulfonate (PSS) with polyacrylamide (PAAm) through a one-step free radical polymerization and chemical cross-linking reaction (Figure 5c). This blend of hydrophilic polymers not only efficiently absorbs and channels water to the water–air interface, facilitating a swift steam generation rate of 2.15 kg·m^−2^·h^−1^ and an energy efficiency of about 97.2%, but the pliability and resilience of the PAAm network also grant the hydrogel remarkable deformability and tensile strength, securing its suitability for use in demanding conditions.

Pros and cons. The customizable properties of synthetic polymer hydrogels can be readily adjusted by controlling the polymer’s composition, molecular weight, chemical structure, crosslinking degree, molar ratio, and external environmental conditions to meet the requirements of interfacial solar-driven evaporation systems. Traditional single network hydrogels cannot fully meet the design standard of ISDE systems, necessitating a shift towards multi-network hydrogel strategies. However, this approach often requires the use of a broader range of raw materials, more complex and refined manufacturing processes, and more challenging large-scale production efforts, resulting in higher production costs.

### 3.2. Hybrid Composite Hydrogels

**Figure 6:**
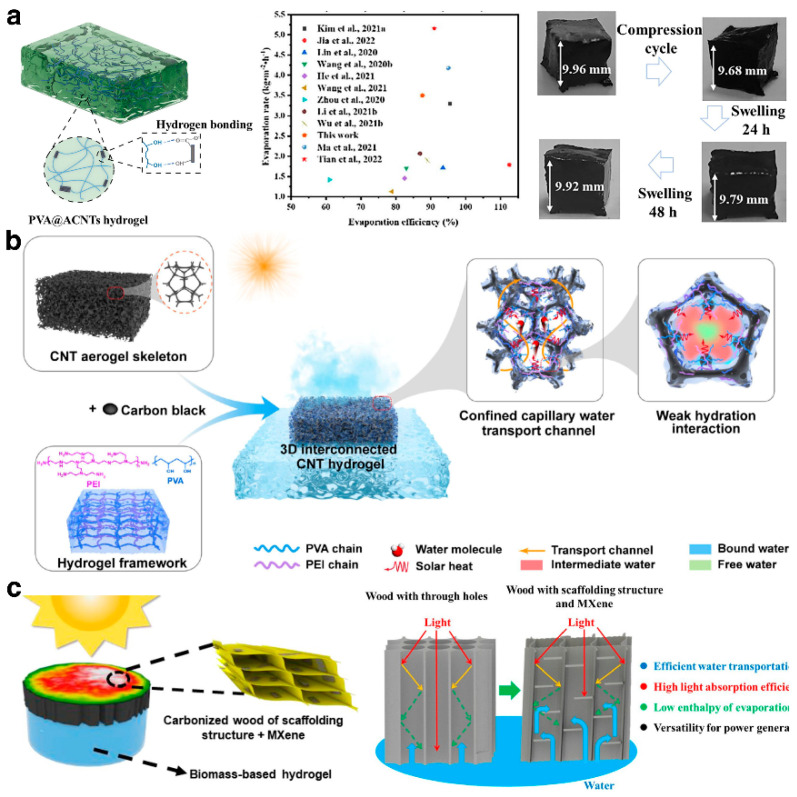
(**a**) The PVA@ACNT composite hydrogel with mechanically robust for high performance solar-driven interface evaporation. Reprinted with permission from ref [87]. Copyright 2023 Elsevier. (**b**) The scheme of interface solar-driven evaporation via the design of a 3D interconnected polymetric network in CNT cellular structure. Reprinted with permission from ref [65]. Copyright 2023 Elsevier. (**c**) Composite biomass hydrogel featuring a scaffold structure of carbonized wood modified by MXene. Reprinted with permission from ref [12]. Copyright 2022 American Chemical Society.

As research into hydrogel technology deepens, composite hydrogels are increasingly gaining attention. Drawing inspiration from rubber reinforcement technology, the development of nanocomposite hydrogels involves the uniform dispersion of nanoscale colloidal particles within a polymer matrix, utilizing the large specific surface area of these nanoparticles to expand the range of surface activity, thereby significantly enhancing the overall performance of the hydrogel [96]. Gao et al. [87] prepared a uniform black PVA/ACNTs mixture by heating and blending acidified carbon nanotubes (ACNTs) with polyvinyl alcohol (PVA), and they constructed a PVA@ACNTs hydrogel-based solar evaporator using a freeze–thaw method (Figure 6a). The porous nature of ACNTs optimized the hydrogel’s porous network, accelerating rapid moisture transfer and steam release during the evaporation process, achieving a high evaporation rate (3.85 kg·m^−2^·h^−1^) and photothermal conversion (87.6%). Additionally, the abundant hydroxyl and carboxyl groups on the surface of the ACNTs formed interface hydrogen bonds with the PVA polymer chains, greatly enhancing the mechanical stability of the hydrogel. On another note, Fan et al. [97] developed a PAAc/SiO_2_-g-PAAm nanocomposite hydrogel using SiO_2_-g-PAAm as a dynamic cross-linking center, which displayed excellent evaporation performance and rapid recovery and self-healing capabilities. Researchers are also developing composite hydrogel substrates by doping them with functional materials like graphene [50] and metal–organic frameworks (MOFs) [42]. They typically employ layer-by-layer self-assembly techniques or chemical cross-linking methods to ensure effective binding and synergistic interaction among the components, significantly boosting the system’s photothermal efficiency and mechanical strength [98]. Furthermore, various porous materials are commonly used as scaffolds in hydrogels to construct multifunctional hydrogels with intricate structures. Zhang et al. [65] utilized cellular carbon nanotubes (CNTs) as a scaffold, in combination with polyvinyl alcohol (PVA), polyethyleneimine (PEI), and carbon black particles (CBs), to develop a hydrogel with a three-dimensional interconnected topological structure (Figure 6b). This hydrogel is characterized by its plentiful hydrophilic capillary nanochannels and weaker water–polymer polar interactions, achieving an impressive water evaporation rate (3.55 kg·m^−2^·h^−1^), and it can effectively generate water in extreme environments. The multifunctional solar evaporator, designed by Pang et al. [12], features a scaffold structure of carbonized wood modified by MXene within a composite biomass hydrogel (Figure 6c). This loose and orderly support structure of the carbonized wood significantly enhances water transport efficiency, boosts capillary forces, and strengthens light absorption by the multiple reflections of light. Moreover, the self-assembled micrometer fiber structure effectively inhibits salt deposition within the scaffold, and the MXene modification in the carbonized wood binds strongly with water molecules, thus lowering the evaporation enthalpy and contact angle.

**Figure 7:**
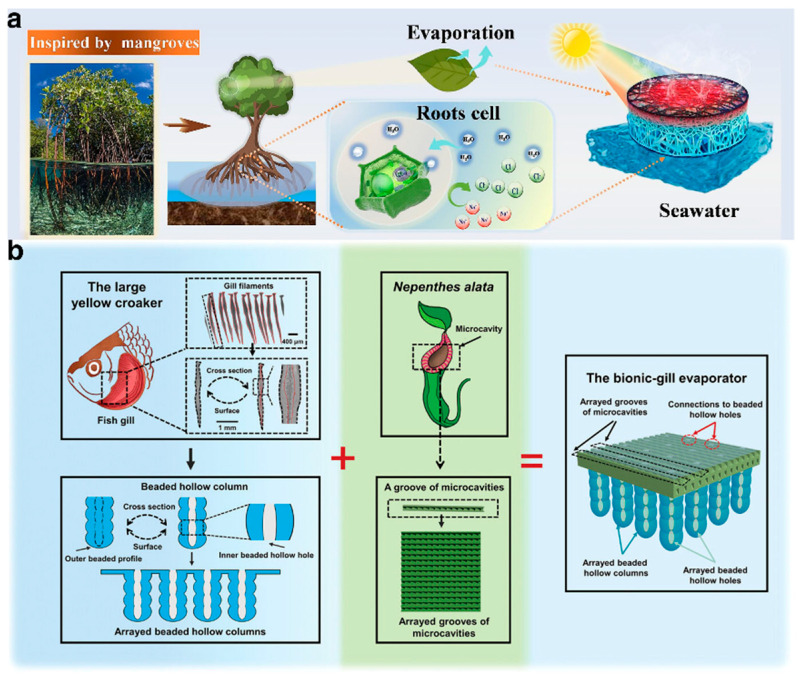
(**a**) A mangrove-inspired composite hydrogel formed by modifying the porous biomass of loofah sponges with sodium polyacrylate. Reprinted with permission from ref [13]. Copyright 2023 Elsevier. (**b**) Inspired by the large yellow croaker and Nepenthes alata, the bionic-gill 3D hydrogel ISDE system was designed with multidirectional crossflow salt mitigation. Reprinted with permission from ref [100]. Copyright 2023 Wiley-VCH Verlag GmbH.

The forms and processes of nature, refined over millions of years, are highly optimized for adaptation to diverse environments [99]. Hydrogels, known for their high controllability, harness endless inspiration and possibilities from the variety found in nature. Inspired by the salt excretion mechanism of mangroves, Bai et al. [13] modified the porous biomass of loofah sponges (LFs) with sodium polyacrylate (PAAS), creating an LF-PAAS composite hydrogel (Figure 7a). This hydrogel is highly hydrophilic, with a tiered large-pore structure and honeycomb-like microchannels that effectively regulate moisture during the solar evaporation process. Additionally, the hydrogel’s negative charge functional groups, COO^−^, in the PAAS network help limit the activity of Na^+^, reducing the diffusion of salt ions to the water supply layer. This feature enables the evaporation system to maintain a consistent water evaporation rate exceeding 1.45 kg·m^−2^·h^−1^ in saline water, across a salinity range of 35–200 g·kg^−1^. Dong et al. [100], inspired by the excellent salt resistance property of the gill filaments of large yellow croakers and the rapid liquid transport property of the peristome surface of Nepenthes alata, designed a bionic-gill 3D hydrogel ISDE system. This system comprises arrayed beaded hollow columns and an upper surface with arrayed grooves of microcavities (Figure 7b). The hydrogel was engineered to facilitate multidirectional crossflow salt ion migration, aiming to achieve a high evaporation rate (2.53 kg·m^−2^·h^−1^) and energy efficiency (99.3%).

Pros and cons. Hybrid composite hydrogels exhibit synergistic effects by combining the advantages of various materials, showcasing rich structural diversity and enabling the integration of multiple functions and properties. Additionally, the inclusion of extra functional materials can interact with the existing hydrogel network or provide a scaffold structure, significantly improving the hydrogel’s mechanical properties. However, synthesizing hybrid composite hydrogels requires careful consideration of material compatibility and reaction condition control, along with the use of diverse materials and extra processing steps. Moreover, integrating different materials in a mixed composite hydrogel could affect its long-term stability, potentially leading to functional degradation or loss over time.

## 4. Applications of Hydrogel-Based ISDE Systems

**Figure 8:**
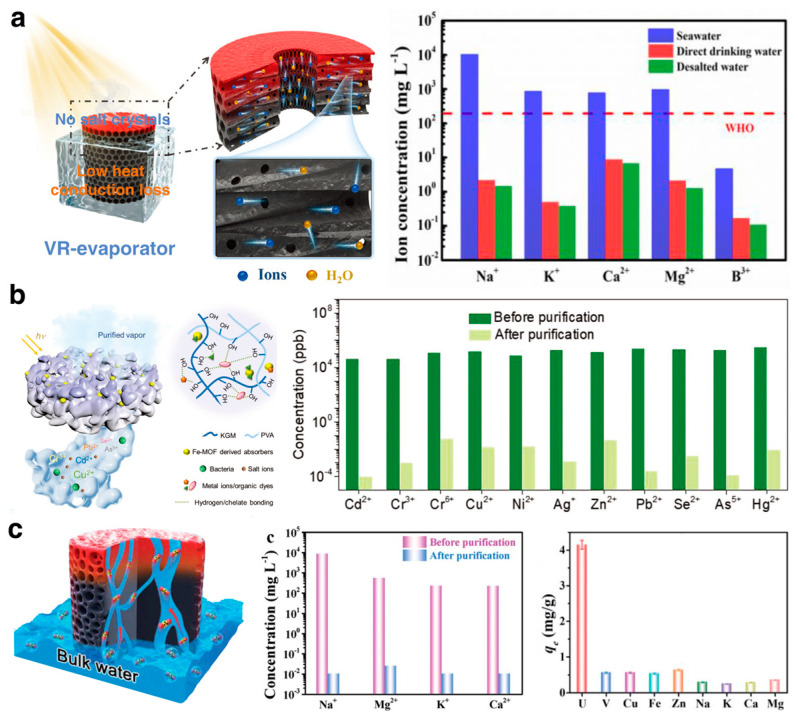
(**a**) The scheme of a hydrogel ISDE system with vertical radiant vessels for efficient desalination performance. Reprinted with permission from ref [69]. Copyright 2022 Wiley-VCH Verlag GmbH. (**b**) A hybrid hydrogel ISDE system for efficient desalination and excellent heavy metal removal. Reprinted with permission from ref [42]. Copyright 2020 Wiley-VCH Verlag GmbH. (**c**) A thiazole-linked COF hydrogel for synergistic seawater desalination and uranium extraction. Reprinted with permission from ref [21]. Copyright 2021 Royal Society of Chemistry.

ISDE systems were initially used for seawater desalination. With the innovative development of soft-elastic hydrogel materials, researchers have extensively explored and validated the immense potential of hydrogel-based ISDE systems in enhancing solar energy utilization, durability, and salt resistance [101,102]. Hu et al. [69] utilized soy protein isolate (SPI) and hydroxyethyl cellulose (HEC) with epoxy cross-linking and freeze-casting techniques to create a 3D hydrogel-based ISDE system with a vertical radiation structure, effectively balancing water transport with minimal heat loss. Even in 20 wt% saline water and after 8 h of continuous exposure, this ISDE system maintained an impressive evaporation rate of 3.53 kg·m^−2^·h^−1^ without salt accumulation (Figure 8a). Yu et al. [54], through a self-assembly templating method (SAT), developed a three-dimensional, hierarchically interconnected porous hydrogel (IPH) that drastically reduced the concentration of salt ions in seawater samples by more than three orders of magnitude. This IPH-based ISDE system maintained stable evaporation for over 100 h without requiring cleaning. Indeed, given the complex biogeochemical nature of real seawater, hydrogel-based ISDE systems encounter significant challenges beyond high salinity. These include a considerable presence of competing metal ions and a notable propensity for marine biofouling [103]. Despite the long road ahead to efficiently extract clean water from real seawater environments, researchers persistently work to surmount these environmental hurdles.

Hydrogel-based ISDE systems broaden their application scope to treat or purify wastewater, relying on adsorption. Yu et al. [42] integrated renewable biomass konjac glucomannan (KGM) with photothermal nanoparticles derived from iron-based metal–organic frameworks (Fe-MOFs) into the PVA network (Figure 8b). This maintains a high evaporation rate in environments with broad pH ranges and high salinity, while effectively removing heavy metal ions and organic dyes through the hydrogel’s abundant hydroxyl groups, employing hydrogen and chelation bonds. In the face of the critical global shortage of clean water resources, the deployment of hydrogel-based ISDE systems has become a breakthrough in the treatment of domestic wastewater and the purification of polluted rivers [104]. These systems are particularly vital in outdoor environments where clean water is scarce, proving to be indispensable in emergency situations [68].

Additionally, with their excellent separation capabilities, hydrogel-based ISDE systems are used not only for obtaining clean water but also for extracting valuable substances from water. For example, nuclear contamination significantly impacts the environment. However, extracting radioactive metals such as uranium can mitigate environmental pollution and encourage resource reuse [105]. Qiu et al. [21] introduced a novel concept of concurrent functionality in both seawater desalination and uranium extraction, reporting a thiazole-linked COF hydrogel that promotes uranium extraction while desalinating seawater (Figure 8c). Yang et al. [106] developed a graphene oxide-based functional chitosan hydrogel, leveraging the high affinity of the phosphate group for uranium and achieving a high adsorption capacity, selectivity, rate, and recyclability for uranium in seawater.

## 5. Conclusions and Perspectives

### 5.1. Conclusions

In recent years, considerable advancements have been made by scientists from various disciplines through joint efforts in the development of ISDE systems (Table 2). This review has methodically outlined the latest advancements in hydrogel-based ISDE systems from three main aspects: the required properties, the preparation methods, and the role played in application scenarios. Initially, we explored the design criteria for hydrogel substrates in ISDE systems aimed at reducing water evaporation enthalpy, enhancing thermal efficiency, stabilizing water supply, and preserving pollution resistance and mechanical stability. These criteria represent innovative strategies to ensure the sustained operation of high-performance ISDE systems. The review then meticulously outlined the fabrication techniques for hydrogel substrates, categorized into synthetic polymeric hydrogels and hybrid composite hydrogels, introducing novel methods that could potentially revolutionize solar evaporation technology. The applications we highlighted include seawater desalination, wastewater treatment, and selective extraction, each demonstrating the practical implications of these innovations. The purpose of this review was to furnish a comprehensive overview of recent research developments in the ISDE field, aiming to inform researchers in related disciplines of the unique benefits that hydrogels offer. It is hoped that the insights gained from this review will contribute to the advancement of ISDE technology towards the realization of its practical applications in hydrogel technology.

### 5.2. Perspectives

Despite the clearly demonstrated advantages of hydrogel-based interfacial solar-driven evaporation and its improved performance, further fundamental studies are necessary to fully elucidate the roles of hydrogels in energy, mass, and momentum transfer during interfacial solar-driven evaporation. This will enable a more profound comprehension of the involved processes, thereby leading to improved performance. In both laboratory and small-scale outdoor settings, the ISDE system has demonstrated notable evaporation rates and energy conversion efficiency with both simulated and real water samples, showcasing commendable salt tolerance and stability. However, more research is needed to bridge the gap between the current state of research and implementation on a larger scale. In contrast to the 20–50 m^2^ effective area of seawater desalination reverse osmosis membrane elements used in commercial large-scale applications, the hydrogel employed for interfacial solar-driven evaporation is currently limited to laboratory scales of 1–100 cm^2^ [64,77,84]. The emergence of hydrogels provides a possibility for the development of ISDE systems, as highly adjustable hydrogels can be used to reduce evaporation enthalpy, control water flow rate, minimize heat loss, resist pollution, enhance stability, etc. Nevertheless, hydrogel-based ISDE systems still face some unresolved challenges, summarized as follows:

(1) There is still scope for enhancement in the evaporation performance of hydrogel-based ISDE systems in actual environments. Currently, the optimal evaporation performance of solar evaporators is mainly measured in stable laboratory conditions, while laboratory testing conditions are not representative of performance in natural sunlight that exhibits a longer wavelength and lower intensity [67,68,103]. How to intelligently adapt to changing environments to maintain sustained high evaporation performance is a pressing issue. Smart hydrogels can exhibit different swelling behaviors in response to external environmental stimuli, offering a new approach for intelligent evaporation using ISDE systems.

(2) At present, research on the lifespan of solar evaporators remains limited. They may initially achieve good evaporation performance, while the rate of performance degradation over time is unclear. It is not clear how long the evaporative materials can be used before replacement is necessary and whether the replaced materials can be reused after simple treatments. These material lifespan issues remain unresolved.

(3) The complex environment requires hydrogel-based ISDE systems to be sufficiently strong or self-repairable to maintain long-term stability. This necessitates elaborate design of the hydrogels, which often means the use of a greater variety of raw materials, more complex and delicate processes, more challenging large-scale manufacturing, and higher costs. This poses another challenging problem for the practical application of ISDE systems.

(4) It is currently unclear whether the clean water obtained from the actual target water body through ISDE systems can be directly used by humans or needs further treatment. For example, in the application of ISDE systems for seawater desalination, not only efficient desalination needs to be considered, but also other pollutants that cannot be ignored, such as low-boiling organic pollutants [67,118,119] and radioactive contaminants [120]. Therefore, ISDE systems are expected to have a more comprehensive ability to remove pollutants in the actual water purification process, rather than just the ability to remove a single category of pollutants.

ISDE systems have experienced rapid development in recent years. It is envisioned that ISDE systems will become one of the key technologies for obtaining clean water. With the unique advantages provided by hydrogels, hydrogel-based ISDE systems will undoubtedly be a research hotspot within ISDE, and they will be extensively developed and applied in the coming years.

## Figures and Tables

**Table 1:** Design criteria of hydrogel substrates in ISDE systems along with relevant studies.

Design Criteria of Hydrogel Substrates	Significance	Ref.
Appropriate hydrophilicity	Reduce the mutual binding of water molecules and increase the proportion of intermediate water.	[47]
	Ensure low evaporation enthalpy and swift water transport.	[24]
Low heat loss	Enhance evaporation efficiency.	[18,19]
Micro-water channels	Manage the water transport for aligning the water transport rate with the evaporation rate.	[49,51]
Pollution resistance	Avoid salt accumulation and maintain stable operation.	[52,53]
Stable mechanical strength	Withstand continuous solar radiation and physical and chemical stresses caused by environmental changes	[54,55,56]

**Table 2:** Evaporation performance of ISDE systems based on various substrates (1 sun).

Type of Substrate	Materials of Substrate	Photothermal Material	Evaporation Rate (kg·m^−2^ h^−1^)	Evaporation Efficiency (%)	Major Function	Additional Functions	Ref.
Hydrogels	Janus ion-selective hydrogel	Carbon particles	6.86	Low-heat loss	Desalination (high-salt)	None	[101]
	Double-network hydrogel	Polypyrrole	4.145	>120	Desalination	None	[51]
	Polyzwitterionic hydrogel	Activated carbon particles	4.14	~94	Desalination	Removal of heavy metal ions	[53]
	Ti_2_O_3_/PVA nanocomposite hydrogel	Ti_2_O_3_ particles	~4.0	93	Desalination	None	[77]
	PVA/ACNTs nanocomposite hydrogel	Acidified carbon nanotubes	3.85	87.6	Desalination	Purification of oil-in-water emulsion	[87]
	Ti_2_O_3_/PVA nanocomposite hydrogel	Ti_2_O_3_ nanoparticles	~3.6	~90	Desalination	None	[61]
	Polyelectrolyte hydrogel	Carbon black	3.52	97.2	Desalination	Thermoelectric power generation	[107]
	P(DMAPS-coNIPAM) zwitterionic hydrogel	Polypyrrole	~3.45	~95	Desalination	None	[62]
	PPy/PVA interpenetrating network hydrogel	Polypyrrole	3.2	~94	Desalination	None	[49]
	PPy/PVA interpenetrating network hydrogel	Polypyrrole	2.64	82.5	Desalination	Removal of chemical oxygen demand	[34]
	PVA/PEDOT:PSS polyelectrolyte hydrogel	Poly(sodium-p-styrenesulfonate)	2.5	90.7	Desalination	None	[52]
	COF-PVA covalently cross-linked hydrogel	Polydopamine	1.5	91.4	Desalination	Uranium extraction capacity	[21]
	Hypercrosslinked polymeric networks hydrogel	Carbon black	1.4	~85	VOCs removal (99.99%)	None	[108]
Other substrates	Cotton towel	Reduced graphene oxide	7.6	Zero energy loss	Desalination	None	[109]
	Glass fiber membrane	Graphene wrapped Fe_3_O_4_ nanoparticles	5.88	/	Desalination	None	[110]
	Cu foam	Nanostructured CuO	4.1	/	Desalination	None	[111]
	Hemp fiber	Ti_3_C_2_Tx (MXene)	3.95	177.8	Desalination	Thermoelectric generation	[112]
	None	Carbonized luffa sponge	3.7	/	Desalination	None	[113]
	PNAGA aerogel	Reduced graphene oxide	3.4	~93	Desalination	Separation performance for oil-in-water emulsions	[114]
	Maize straw aerogel	Nitrogen-doped graphene	3.22	95	Desalination	Purification of organic pollutants and oil-in-water emulsion	[115]
	Porous wood	Polydopamine	2.7	86	Desalination	Removal efficiency for harmful ions and organic pollutants	[116]
	Nylon	Polypyrrole	2.62	92.7	Desalination	None	[117]

## Data Availability

Not applicable.

## Nomenclature

**Nomenclature**			
*r*	evaporation rate	*q_in_*	total solar energy received
*η*	evaporation efficiency	Δ*m_sunlight_*	apparent evaporation rate
Δ*m*	mass of water vapor generated through evaporation	Δ*m_dark_*	evaporation rate in the dark
*S*	effective evaporation area	*h_vap_*	total enthalpy change of water transitioning from liquid to gas
*t*	time of irradiation	*C_opt_*	optical concentration on the absorber’s surface
*q_evap_*	evaporation energy	*P*_0_	standard solar radiation, set at 1 kW·m^−2^
**Abbreviations**			
ISDE	interfacial solar-driven evaporation	PEI	polyethyleneimine
PVA	polyvinyl alcohol	CB	carbon black
OTS	trichloro(octadecyl)silane	LF	loofah sponge
COF	covalent organic framework	MXene	Ti_3_C_2_T_x_
rGO	reduced graphene oxide	SPI	soy protein isolate
SA	sodium alginate	HEC	hydroxyethyl cellulose
PAAS	sodium polyacrylate	SAT	self-assembly templating method
PSS	polystyrene sulfonate	IPH	interconnected porous hydrogel
PAAm	polyacrylamide	KGM	konjac glucomannan
ACNTs	acidified carbon nanotubes	PPy	polypyrrole
MOFs	metal–organic frameworks	PEDOT	poly (3,4-ethylenedioxythiophene)
CNT	cellular carbon nanotube	PNAGA	poly (N-acryloyl glycinamide)
